# Method for Improving Positioning Accuracy of Rotating Scanning Satellite Images via Multi-Source Satellite Data Fusion

**DOI:** 10.3390/s26030850

**Published:** 2026-01-28

**Authors:** Liwei Wang, Peng Wang, Yamin Zhang, Yi Wang, Bo Chen

**Affiliations:** 1 School of Aerospace, Harbin Institute of Technology, Shenzhen 518055, China; 23s061021@stu.hit.edu.cn (L.W.); zhangyamin@stu.hit.edu.cn (Y.Z.);; 2 Key Laboratory of Aerospace RS Big-Data Intelligent Processing and Application of Guangdong Higher Education Institutes, Harbin Institute of Technology (Shenzhen), Shenzhen 518055, China; 3 Space Engineering University, Beijing 101416, China

**Keywords:** geometric positioning, joint adjustment, rotating scanning, RPC model

## Abstract

**Highlights:**

**What are the main findings?**

- A multi-source collaborative positioning framework integrates ZY-3 and GF-2 imagery to achieve a planar accuracy of 4.01 m and an edge matching RMSE of 2.52 m.
- The proposed grid-based feature extraction and joint adjustment method successfully attains meter-level positioning accuracy (4.68 m and 5.22 m) for simulated ultra-wide rotating scanning imagery.

**What are the implications of the main findings?**

- The approach effectively mitigates complex geometric distortions in rotating scanning systems without dense ground control points or strict physical models.
- This study provides a robust solution for generating seamless, high-precision orthophoto products from ultra-wide swath satellite data using multi-source fusion.

**Abstract:**

Rotating scanning systems are capable of acquiring ultra-wide swath satellite imagery, but they suffer from significant positioning accuracy degradation due to complex geometric distortions and the difficulty of obtaining ground control points (GCPs) over vast areas. To address these issues, this paper proposes a precise positioning method based on multi-source satellite data fusion. By comprehensively utilizing high-resolution images from ZY-3 and GF-2 satellites alongside DEM data, we establish a framework that integrates grid-based feature point extraction, high-precision matching, and multi-image joint adjustment. Specifically, we introduce a matching strategy combining geometric constraints with Least Squares Minimization (LSM) and a robust joint adjustment model to suppress geometric distortions. Experimental validation was conducted using a dataset covering the Beijing area. The results demonstrate that after joint adjustment, the planar accuracy of the imagery reached 4.01 m, and the edge matching Root Mean Square Error (RMSE) between adjacent images was 2.52 m. Furthermore, the cooperative positioning accuracy for segmented simulation data achieved 4.68 m in mountainous areas and 5.22 m in plain areas, meeting the requirements for meter-level positioning. These results verify the effectiveness of multi-source cooperative adjustment in correcting geometric distortions and significantly improving the positioning accuracy of rotating scanning imagery.

## 1. Introduction

With the expansion of remote sensing applications, requirements for information acquisition have evolved remarkably. Critical applications, such as global change monitoring [1] and natural resource surveys, now demand a dual capability: rapid, large-area coverage (i.e., wide swath) combined with high spatial resolution [2]. In recent years, high-resolution observation technology has advanced rapidly, providing rich information resources for the geosciences [3]. However, constraints inherent to existing imaging mechanisms—such as camera fields of view and satellite orbital regression cycles—create a mutual trade-off between spatial resolution and swath width in optical remote sensing satellites [4]. Consequently, simultaneously achieving meter-level resolution and thousand-kilometer-level coverage with a single satellite remains a formidable challenge [5].

To surmount these limitations, the rotating scanning imaging system has been proposed. Unlike the mainstream static pushbroom imaging employed by most optical satellites, the CMOS sensor in a rotating scanning system is aligned along the flight direction but rotates continuously 360° around an axis perpendicular to the trajectory. This mechanism allows the satellite to sweep the ground continuously, acquiring image strips that are subsequently stitched into a seamless panoramic view of the target area.

However, satellite remote sensing imagery obtained via this imaging method faces several key challenges. First, the imaging geometry results from the complex coupling between the satellite’s orbital motion and the high-speed rotation of the sensor, introducing high-order and time-varying geometric distortions. Such dynamic deformation renders traditional static geometric models and standard Rational Polynomial Coefficient (RPC) correction methods inadequate for achieving high-precision geolocation, often leading to model instability. Second, rotating-scanning satellites typically cover ultra-wide swaths, often on the order of thousands of kilometers [2], making it practically impossible to obtain a sufficient number of well-distributed, field-surveyed ground control points (GCPs) across the entire coverage area. To bridge this gap, this paper proposes a multi-source collaborative positioning framework that leverages existing high-precision satellite data (e.g., ZY-3) as a control skeleton to automate accuracy transfer, bypassing the GCP acquisition bottleneck.

With these research challenges and opportunities, this study aims to propose a positioning framework via multi-source satellite data fusion. The main contributions of this paper are as follows:

1. Establishment of a Positioning Framework via Multi-Source Satellite Data Fusion: We have constructed a comprehensive framework for the precise positioning of ultra-wide swath imagery. By synergistically utilizing multi-source high-resolution optical satellite imagery and DEM data, we established a joint adjustment model that proficiently facilitates error compensation and accuracy transfer across heterogeneous data sources.
2. Development of High-Precision Tie Point Extraction Technology: Addressing the severe deformation characteristics of ultra-wide images, we developed a robust automatic tie point extraction technique. This method integrates an image blocking and gridding strategy with geometric constraints and Least Squares Minimization (LSM) optimization, ensuring a balanced distribution and high-precision extraction of feature points.
3. Comprehensive Experimental Validation: We designed a rigorous validation scheme using simulated wide-swath data derived from real satellite imagery. Through quantitative evaluation of both absolute positioning accuracy and relative edge matching accuracy, we verified the method’s effectiveness and engineering practicality for rotating scanning satellite applications.

This study presents significant advancements in the geometric rectification of spaceborne sensors, primarily through two key contributions: (1) To address the critical challenge of acquiring Ground Control Points (GCPs) in ultra-wide swath imaging, an automated control information transfer mechanism utilizing multi-source reference imagery has been developed, thereby overcoming the technical bottleneck associated with control data scarcity for swaths spanning thousands of kilometers. (2) Targeting the severe geometric instability inherent in rotating scanning sensors, a joint adjustment model is proposed to successfully suppress kilometer-level distortions. By specifically modeling and compensating for high-order time-varying errors, this model achieves a substantial reduction in absolute positioning error from the kilometer-scale commonly reported in the literature to within 10 m.

## 2. Related Work

The geometric processing of optical satellite imagery has evolved notably, driven by the diversity of imaging mechanisms and the demand for high-precision geospatial products. This section reviews the current state of research in three key areas relevant to this study: geometric modeling of whiskbroom/scanning systems, jitter detection and correction, and advanced computational processing for remote sensing data.

### 2.1. Geometric Modeling and Calibration of Whiskbroom Imaging Systems

While geometric correction for static pushbroom cameras is relatively mature [6,7], the modeling of whiskbroom scanning systems presents unique challenges due to the complex coupling of scanning motion and satellite flight. Early research by Breuer and Albertz established foundational concepts for airborne whiskbroom scanners, emphasizing the need for hybrid auxiliary data to correct panoramic and motion-induced distortions [8]. Building on these principles, Uto et al. developed low-cost whiskbroom imagers using optical fiber bundles, demonstrating that hardware-specific geometric distortions can be mitigated through precise optical coupling and registration algorithms [9].

In the context of spaceborne platforms, the “rotating scanning” or “conical scanning” mechanism has been increasingly explored to achieve ultra-wide swaths. Wang et al. proposed a conceptual rotational mode for optical conical scanning imaging small satellites, analyzing the imaging degradation caused by the compound motion of satellite flight and sensor rotation [10]. To mitigate image blur in such dynamic systems, Sun et al. introduced an image motion compensation method using two-axis fast steering mirrors, establishing a quantitative relationship between the scanning trajectory and compensation rates [11].

Specific missions have driven further methodological innovations. For the SDGSAT-1 Thermal Infrared Spectrometer (TIS), Hu et al. detailed the system design which utilizes a 1-D scanning mirror to achieve a 300 km swath [12], while Li et al. proposed a three-step in-orbit calibration strategy decoupling errors from exterior orientation and scanning compensation parameters [13]. Similarly, for the HJ-2 A/B satellites, Zhang et al. introduced a multi-focal-plane-array joint calibration method to improve band-to-band registration accuracy [14]. Additionally, Sun developed a geometric calibration model for linear-array whiskbroom satellites based on look-angle corrections, utilizing cubic polynomial surfaces to fit correction quantities [15].

Most relevant to this study, Liu et al. constructed a rigorous imaging model specifically for linear array sensors performing circular scanning perpendicular to the orbit, proposing a correction method based on orbital attitude parameters and spline function fitting [16]. Furthermore, Li et al. applied rigorous photogrammetric processing to Tianwen-1 HiRIC imagery, demonstrating that bundle adjustment with high-frequency jitter compensation can achieve sub-pixel accuracy even in complex deep-space environments [17]. Zhang et al. also developed a calibration method for airborne linear-array multi-camera systems, utilizing bundle adjustment with orientation constraints to ensure high-precision stitching [18].

Despite these advances in imaging models, existing methods primarily focus on sensor-specific calibration or standard linear scanning mechanisms. They often lack a unified solution for the complex, high-order geometric distortions characteristic of continuous 360° rotating scanning systems over ultra-wide swaths. To address this, this study builds upon these rigorous geometric principles, proposing a refined model capable of integrating external constraints to suppress the specific non-linear deformations inherent in rotating scanning imagery.

### 2.2. Attitude Jitter Detection and Correction

For high-resolution satellites, micro-vibrations or “jitter” can remarkably deteriorate geometric quality. Rotating scanning systems are particularly susceptible to high-frequency attitude variations during the scan cycle. Zhang et al. addressed this by deriving an image-based jitter inversion model that accounts for the Time Delay Integration (TDI) effect [19]. Additionally, Zhang et al. conducted a comprehensive simulation of vibration influences on rotating scanning satellites, analyzing how different vibration frequencies affect geometric positioning and providing a theoretical basis for payload structural design [20].

However, while these studies successfully characterize vibration impacts, practical correction of these high-frequency attitude variations remains challenging, especially in scenarios lacking dense GCPs. Therefore, distinct from purely internal jitter detection, this paper proposes to mitigate these geometric instabilities by incorporating multi-source reference data into a joint adjustment framework, thereby compensating for positioning errors induced by dynamic sensor motion.

### 2.3. Multi-Source Fusion and Advanced Geometric Processing

The synergy of multi-source data is critical for improving mapping accuracy. Silva et al. demonstrated a fusion framework combining GEDI, ICESat-2, and NISAR data, proving that integrating sparse lidar samples with wall-to-wall radar data can yield spatially comprehensive biomass maps [21]. To reduce dependence on ground control points (GCPs), Afsharnia et al. proposed a geometric correction method based on DEM matching, correcting orbital parameters without ground control [22]. Similarly, Tatar introduced a geolocation bias correction for Cartosat-1 using virtual GCPs generated from open-source DEMs [23]. Zhou et al. achieved high-accuracy georeferencing for GF-6 WFV images using a similar reference-based approach [24].

In terms of advanced processing, deep learning and implicit neural representations are reshaping geometric pipelines. Song et al. evaluated deep learning-based features against handcrafted features for satellite image matching, finding that learning-based methods offer superior robustness [25]. Lu et al. proposed SatMVS, transferring Multi-View Stereo (MVS) neural networks to satellite imagery for robust height estimation [26]. Furthermore, Marí et al. explored the application of Neural Radiance Fields (NeRF) to multi-date Earth observation data, demonstrating that implicit representations can model geometry and appearance changes effectively [27]. Finally, Liu et al. proposed a parallel optimization approach on DCU clusters to handle the massive data volume generated by wide-swath satellites [28].

Nevertheless, directly applying these general fusion strategies to rotating scanning systems is hindered by the substantial differences in resolution and viewing geometry compared to standard reference satellites (e.g., ZY-3, GF-2). Bridging this gap, our work develops a robust multi-source fusion pipeline. While learning-based methods mentioned above show promise in general scenarios, they often require extensive training on domain-specific datasets (e.g., rotating scanning distortions) and can lack the rigorous geometric interpretability required for metric-level joint adjustment. Therefore, this study prioritizes a classical yet rigorous photogrammetric approach to ensure deterministic sub-pixel precision.

## 3. Methods

**Figure 1:**
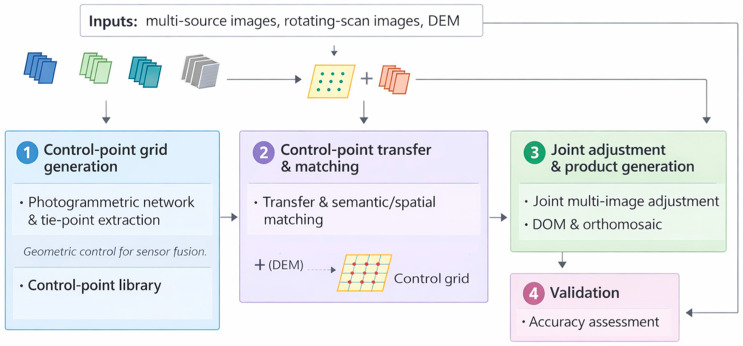
Overview of the workflow for multi-source collaborative positioning.

This study employs an integrated collaborative positioning framework to rectify the geometric distortions of rotating scanning satellite imagery by fusing multi-source high-resolution data (Figure 1). A high-precision control grid is first constructed by extracting feature points from reference ZY-3 and GF-2 imagery using a grid-based strategy and determining their 3D coordinates via a reference DEM. To address the radiometric and geometric disparities between heterogeneous sensors, a coarse-to-fine matching approach combining Normalized Cross-Correlation (NCC) and Least Squares Matching (LSM) is utilized to generate reliable tie points. Subsequently, for the target rotating scanning images, a Multi-Directional Spatial Context Information (MSCI) matching method is applied to accurately transfer control information. Finally, a multi-image joint adjustment model, reinforced by robust estimation, is implemented to optimize the orientation parameters and generate high-precision Digital Orthophoto Maps (DOM).

### 3.1. Multi-Source Control Point Grid Generation Method

#### 3.1.1. Geometric Control Principle of Heterogeneous Sensor Integration

Integrating data from diverse platforms (e.g., GF-1, GF-2, ZY-3) presents inherent challenges due to differences in spatial resolution, spectral response, and initial positioning accuracy. To address the aforementioned challenges, this section employs a heterogenous sensor integration strategy centered on constructing a hierarchical, robust joint adjustment system. Its fundamental principle is as follows: first, all images to be fused are treated as a unified photogrammetric regional network. Second, high-precision connection point extraction establishes geometric links between all overlapping image pairs. Finally, within a unified adjustment model, the systematic error model parameters for all images and the three-dimensional ground coordinates for all connection points are jointly solved. In this progress, we adopt a “hierarchical constraint” strategy within a unified photogrammetric block adjustment. The core principle is to assign higher weights to images with superior initial positioning accuracy (such as ZY-3), allowing them to function as a “control skeleton”. Images with lower initial accuracy are then geometrically constrained to these anchors. Through this mechanism, geometric accuracy is proficiently transferred and distributed throughout the entire block, unifying the geometric datum across the dataset.

#### 3.1.2. High-Precision Tie Point Extraction Strategy

We employ a robust two-step matching strategy: “Normalized Cross-Correlation (NCC) Coarse Matching” followed by “Least Squares Matching (LSM) Fine Matching”.

NCC Coarse Matching To mitigate the effects of geometric deformation and radiometric differences, NCC is used for initial matching. The NCC coefficient is calculated as follows:

(1) $$C=\frac{\sum_{i=1}^{m}\sum_{j=1}^{n}\left[f(i,j)-\bar{f}\right]\left[g(i,j)-\bar{g}\right]}{\sqrt{\sum_{i=1}^{m}\sum_{j=1}^{n}{\left[f(i,j)-\bar{f}\right]}^{2}\sum_{i=1}^{m}\sum_{j=1}^{n}{\left[g(i,j)-\bar{g}\right]}^{2}}},$$

where m,n represents the dimensions of the template window, f(i,j) and g(i,j) denote the pixel gray values at corresponding positions in the template and search images, respectively, and $\bar{f}$ and $\bar{g}$ represent the average gray values of the two windows. The location yielding the maximum NCC coefficient is thus regarded as the approximate position of the corresponding point.

To enhance efficiency and robustness against rotation, we implement two optimization strategies:

- Pyramid Strategy: Matching initiates at the top level of a Gaussian pyramid (low resolution) and progressively refines to the bottom level, considerably reducing the search space.
- Rotation Template Generation: Using the RPC files, we estimate the relative rotation angle θ between images. The template is then rotated to compensate for deformation:
  (2) $$\left[\begin{array}{c} x^{\prime }\\ y^{\prime } \end{array}\right]=\left[\begin{array}{cc} \cos\theta & -\sin\theta \\ \sin\theta & \cos\theta \end{array}\right]\left[\begin{array}{c} x-x_{0}\\ y-y_{0} \end{array}\right]+\left[\begin{array}{c} x_{0}\\ y_{0} \end{array}\right],$$
  where (x,y) represents the original template coordinates; $\left(x^{\prime },y^{\prime }\right)$ represents the rotated coordinates; and $\left(x_{c},y_{c}\right)$ represents the rotation center.

LSM Fine Matching: For sub-pixel accuracy, LSM is employed to solve for geometric and radiometric parameters. The fundamental observation equation is:

(3) $$\begin{array}{l} x-x_{0}=-f\frac{a_{1}(X-X_{S})+b_{1}(Y-Y_{S})+c_{1}(Z-Z_{S})}{a_{3}(X-X_{S})+b_{3}(Y-Y_{S})+c_{3}(Z-Z_{S})}\\ y-y_{0}=-f\frac{a_{2}(X-X_{S})+b_{2}(Y-Y_{S})+c_{2}(Z-Z_{S})}{a_{3}(X-X_{S})+b_{3}(Y-Y_{S})+c_{3}(Z-Z_{S})} \end{array}$$

where (X,Y,Z) are the object space coordinates; $\left(X_{S},Y_{S},Z_{S}\right)$ denote the sensor’s perspective center; f is the effective focal length; and $a_{i},b_{i},c_{i}$ are the elements of the rotation matrix R determined by the platform’s roll, pitch, and yaw.

We utilize an affine transformation model to describe the geometric relationship between windows, solving for 6 geometric parameters and 2 radiometric parameters. The linearized error equation for each pixel is

(4) $$v=g_{x}da+g_{x}xdb+g_{x}ydc+g_{y}dd+g_{y}xde+g_{y}ydf+h_{0}+h_{1}g(x,y)-\Delta g$$

This system is solved iteratively using the least squares method, $X={\left(A^{T}PA\right)}^{-1}A^{T}PL$, until the parameter corrections fall below a preset threshold.

#### 3.1.3. Control Point Library Generation via Moravec Operator

To generate the control point library, we utilize the Moravec corner detection operator [24]. While more modern detectors like Harris, SIFT, or learning-based methods offer higher rotation invariance, the Moravec operator is selected for this framework due to its notably lower computational complexity and high processing speed—critical for handling the ultra-wide swath data involved in rotating scanning imagery. This involves computing the Sum of Squared Differences (SSD) in four directions (horizontal, vertical, diagonal, and anti-diagonal):

(5) $$V=\sum (f(x_{i},y_{i})-f(x_{i}+u,y_{i}+v){)}^{2}$$

The “Interest Value” for each pixel is determined by the minimum SSD among the four directions: $IV=\min(V_{1},V_{2},V_{3},V_{4})$. Following non-maximum suppression to retain only local maxima, the extracted corners are mapped to object space coordinates using the reference DOM and DSM, forming a high-precision control information library.

### 3.2. Collaborative Positioning Based on Semantic Features

Once the control library is established, the next phase is the collaborative positioning of the target rotating scanning imagery.

#### 3.2.1. Control Point Transfer Using Spatial Context Features

Due to the complex geometric distortions and non-linear radiometric differences between the reference and rotating scanning images, traditional intensity-based matching is often insufficient. We therefore propose a matching method based on Multi-Directional Spatial Context Information (MSCI). Instead of using raw pixel values, this method aggregates the gray value relationships between a central pixel and its neighbors to construct structural features, enhancing robustness against contrast variations.

**Figure 2:**
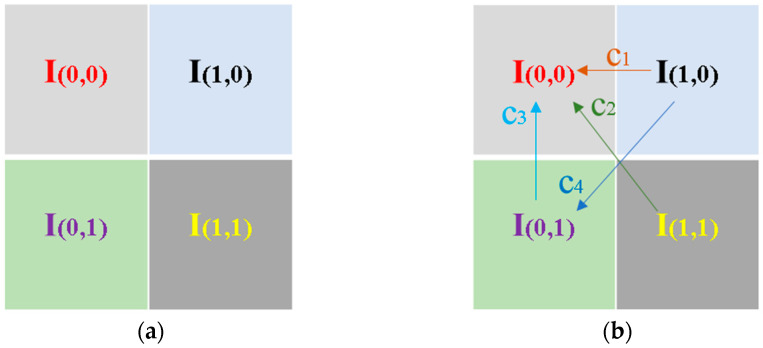
Diagram of Spatial Relationships Between Pixels. (**a**) Schematic diagram of original pixel locations. (**b**) Schematic diagram of spatial contextual information in four directions.

As depicted in the schema in Figure 2, the local neighborhood around a feature point is partitioned into multiple cells. This method aggregates the gray value relationships between a central pixel I(0,0) and its neighbors (I(1,0),I(1,1),I(0,1)) to construct structural features. The spatial relationship and intensity contrast between these cells are visualized in Figure 2.

By linking each directional cell in Figure 2 to a specific dimension in the formula, the MSCI model ensures that the matching cost calculated in Equation (3) reflects the actual spatial structure of the satellite imagery.

To suppress noise inherent in multi-source data (e.g., SAR or LiDAR intensity), Gaussian filtering is applied to the feature channels. Subsequently, the features are normalized to create the MSCI descriptor, enhancing robustness against contrast variations:

(6) $$MSCI(x)=\exp\left(-\frac{\|C(x)-C(y){\|}^{2}}{\sigma^{2}}\right)$$

Finally, matching is performed in the frequency domain. By calculating the Cross-Power Spectrum via Fast Fourier Transform (FFT), we obtain a pulse function where the peak location indicates the precise translation shift.

#### 3.2.2. Multi-Image Joint Adjustment and Robust Estimation

We employ a multi-image joint adjustment model based on collinearity condition equations to optimize the orientation parameters. The process includes extracting tie points between overlapping strips and transferring control points from the library [24]. Error equations are constructed for both control points (using affine parameters as unknowns) and tie points (using object coordinates as unknowns). To eliminate gross errors generated during automatic matching, we apply robust estimation using the Iteratively Reweighted Least Squares (IRLS) method. The weight matrix is updated in each iteration using the Huber weight function:

(7) $$P_{ii}=\left\{\begin{array}{ll} 1 & |v_{i}|\leq k\sigma_{0}\\ k\sigma_{0}/|v_{i}| & |v_{i}|>k\sigma_{0} \end{array}\right.$$

This ensures that observations with large residuals are down-weighted, preventing them from distorting the final adjustment solution.

## 4. Results

### 4.1. Experiment Area and Data Sources

To validate the effectiveness of the proposed method across different geomorphological conditions, the test area was selected as the experimental site (N 39.465° to N 40.461°, E 115.672° to E 116.496°). This region provides a natural laboratory for comparative analysis due to its distinct topographic dichotomy: the northwestern part is dominated by rugged mountainous terrain, while the southeastern part consists of the flat North Plain.

The experimental dataset consists of six multi-source high-resolution optical satellite images:

- Reference Data: Two scenes of ZY-3 panchromatic imagery (2.1 m resolution) and four scenes of GF-2 satellite imagery with high internal geometric stability served as the control backbone;
- Target Data: Rotating scanning satellite simulation data from the research by Xue [1]. To simulate the rotating scanning imagery, the original GF-2 push-broom data was re-projected onto a cylindrical surface. We applied a transformation based on the scanning angle $\theta (t)=\omega \cdot t+\delta_{\theta }$, where ω is the nominal scan rate and $\delta_{\theta }$ represents simulated attitude jitter. This ensures the resulting imagery contains the characteristic S-shape distortion and scale variation typical of rotating sensors. For this comparative study, 80 representative chips were selected: 40 chips covering the western mountainous areas and 40 chips covering the southeastern plains;
- Auxiliary Data: SRTM DEM data were utilized to provide elevation constraints for the joint adjustment and orthorectification processes.

### 4.2. Control Point Extraction and Joint Adjustment Accuracy

**Figure 3:**
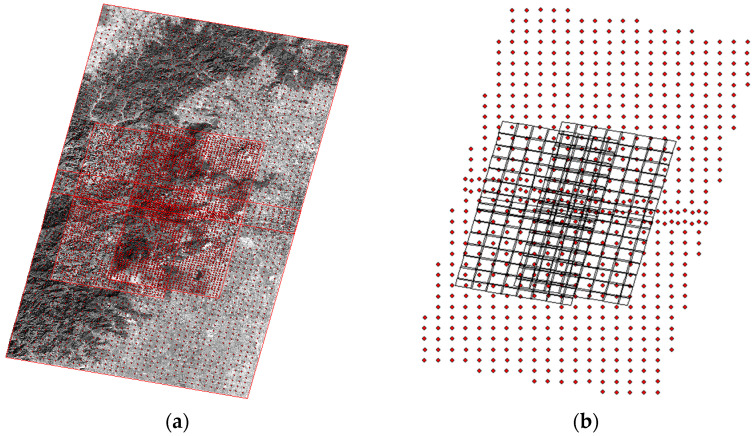
Schematic diagram of generated connection points and control points. (**a**) Distribution map of extracted tie points. (**b**) Distribution map of control points.

Using the grid-based feature extraction and MSCI matching strategy, a dense network of control and tie points was established. As illustrated in Figure 3, the study area is divided into regular grids, and tie points are extracted within each grid to ensure an even spatial distribution. This distribution serves as the physical basis for the ‘control skeleton’ mentioned above, ensuring that the high-precision constraints from ZY-3 are uniformly propagated through the adjustment equations discussed in Section 3.2.2. On average, 2046 points per chip were extracted for the mountainous region and 2475 points per chip for the plain region. The higher point density in the plain region is attributed to the abundance of man-made structures (roads and building corners), which are conducive to feature matching.

Following the multi-image joint adjustment, we evaluated the positioning accuracy using high-precision checkpoints. Table 1 summarizes the positioning accuracy after multi-image joint adjustment. The results demonstrate that the systematic errors across the heterogeneous sensors were efficiently unified, achieving a consistent planar accuracy level. The average planar positioning accuracy (RMSE) after adjustment reached 4.01 m. Considering the reference imagery itself has a planar accuracy of approximately 5 m (leading to a theoretical error propagation of 6.41 m), the adjustment result of 4.01 m significantly outperforms the theoretical value, demonstrating the efficacy of the error compensation model.

Furthermore, we assessed the relative consistency between adjacent images. As shown in Table 2, the average edge matching RMSE between all overlapping image pairs was 2.52 m. This high level of consistency indicates that systematic errors between multi-source images were successfully eliminated, satisfying the requirements for seamless stitching.

### 4.3. Collaborative Positioning Performance on Ultra-Wide Images

The core of the experiment involved evaluating the cooperative positioning accuracy of the simulated ultra-wide rotating scanning image segments. We conducted a rigorous comparison between the original direct positioning and the proposed collaborative adjustment for both mountainous and plain sub-regions.

To compare the model’s performance in mountainous and plain regions, the data for these areas will now be processed and analyzed separately. Table 3 presents the initial direct positioning accuracy of the images from mountainous areas, with Table 4 for plain areas. Due to the high-order distortions inherent in the rotating scanning mechanism, the original imagery exhibited severe geometric displacement.

The initial errors were heavily dominated by the X-direction (cross-track/scanning direction), reaching over 80 m in mountainous areas. This is consistent with the physical characteristics of rotating scanning, where scanning non-linearity and terrain-induced relief displacement are most pronounced in the cross-track direction. Mountainous regions exhibited higher original errors (82.22 m) compared to plains (78.87 m) because the extreme elevation changes further amplified the relief displacement in the scanning geometry.

After applying the proposed collaborative positioning correction based on multi-source control data, the accuracy improved drastically. As detailed in Table 5 and Table 6, the experimental data reveals several key insights:

1. Effective Geometric Recovery: The planar accuracy (RMSE XY) was reduced to under 5.5 m for all regions, meeting the requirements for meter-level positioning.
2. Terrain Adaptability: Interestingly, the correction accuracy in mountainous areas (4.68 m) was slightly superior to that in plain areas (5.22 m). This demonstrates that the MSCI descriptor and DEM-assisted adjustment are highly effective at capturing stable topographic structural features in rugged terrain, which are less susceptible to temporal land-use changes.
3. Error Isotropy: Following the adjustment, the extreme X-direction bias was eliminated. The residual errors in the X and Y directions are now within the same order of magnitude (3–4 m), indicating that the systematic scanning distortions were successfully modeled and suppressed.

### 4.4. Digital Orthophoto Map (DOM) Generation

**Figure 4:**
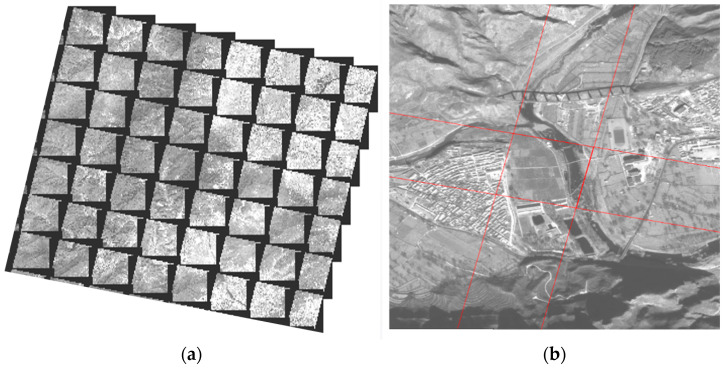
Multi-image registration correction results. (**a**) Coverage map of segmented orthophotos. (**b**) Edge matching detail of segmented orthophotos.

Based on the high-precision orientation parameters obtained from the joint adjustment and the external DEM, we generated a seamless Digital Orthophoto Map (DOM) for the entire experimental area using pixel-by-pixel rectification. Visual inspection of the generated DOM products reveals clear image textures and accurate planar positions (Figure 4). The seamless mosaic further validates the high edge-matching accuracy achieved, proving the method’s engineering practicality for producing high-quality mapping products from rotating scanning satellite data.

Visual inspection of the generated DOM products reveals clear image textures and accurate planar positions. The seamless mosaic further validates the high edge-matching accuracy achieved, proving the method’s engineering practicality for producing high-quality mapping products from rotating scanning satellite data.

### 4.5. Computational Cost and Operational Applicability

To evaluate the operational feasibility of the proposed framework, the processing time was recorded on a Dell 7040 workstation equipped with two Intel Xeon Gold 5118 processors (2.3 GHz) and 64 GB of RAM. For a typical image chip, the automated grid-based feature extraction and MSCI matching require approximately 45.2 s, while the joint adjustment with robust estimation converges in less than 2.5 s. The total processing time for the ultra-wide swath simulation (80 chips) was approximately 64 min. This efficiency indicates that the method is suitable for large-scale, near-real-time satellite data processing without manual intervention.

## 5. Discussion

The results of this study demonstrate that the proposed multi-source data fusion framework proficiently addresses the geometric challenges of rotating scanning satellites. A critical interpretation of the results reveals a counter-intuitive finding: despite the higher initial geometric complexity in mountainous areas (82.22 m initial error), the final collaborative positioning accuracy (4.68 m) is superior to that of the plain areas (5.22 m). This disparity highlights the relative contribution of the MSCI descriptor. In urban plains, high-frequency temporal changes in buildings and shadows introduce ‘textural noise’ that slightly degrades matching precision. In contrast, the rugged terrain provides stable, unique topographic skeletons (e.g., ridges and valleys) that the MSCI descriptor captures with higher semantic fidelity. This suggests that the proposed method is particularly robust for remote, unpopulated mountainous regions where GCPs are hardest to obtain.

Despite these promising results, several avenues for improvement remain:

1. Integration of On-board Data: Future work should incorporate satellite attitude and Inertial Measurement Unit (IMU) data to construct a more rigorous physical imaging model, thereby reducing error accumulation at the source.
2. Robustness in Complex Terrain: While the method performed well in the test area, extracting reliable tie points in extreme terrains (e.g., high relief mountains) or urban canyons remains challenging. Developing adaptive filtering and matching algorithms for these scenarios is necessary.
3. Multi-Modal Fusion: To further enhance positioning capabilities, particularly in varying weather conditions, we aim to explore the online collaborative processing of heterogeneous data, including Optical, SAR, and LiDAR, to achieve intelligent, near-real-time earth observation processing.
4. Transferability and Limitations in Other Terrains: While the experimental results in demonstrate high accuracy across mountainous and plain areas, the transferability of the proposed method to other global terrains warrants further discussion:
  - High-Relief Mountains: The synergy between MSCI semantic features and DEM-based elevation constraints allows the model to proficiently rectify non-linear relief displacements. However, in extreme high-relief areas (e.g., the Himalayas), excessive radar/optical shadowing or snow cover may reduce the number of available tie points, potentially requiring higher-resolution reference DEMs to maintain sub-pixel accuracy.
  - Deserts and Low-Texture Regions: A primary limitation arises in extremely homogeneous landscapes such as vast sand deserts or consistent ice sheets. Because the MSCI descriptor relies on local spatial contextual structures, the absence of distinct textural gradients in these regions would lead to sparse or unreliable feature matching. In such cases, the framework would rely more heavily on the satellite’s initial ephemeris data or would require the integration of multi-modal data (e.g., SAR intensity features) which are less dependent on optical texture.
  - Dependency on Reference Quality: The final absolute positioning accuracy is intrinsically capped by the precision of the reference ‘skeleton’ (e.g., ZY-3 imagery). In remote regions where high-precision reference imagery or GCP-calibrated base maps are unavailable, the absolute accuracy may degrade toward the level of the target satellite’s raw orientation parameters.

## 6. Conclusions

This study established a collaborative positioning framework for ultra-wide rotating scanning satellite imagery. By utilizing ZY-3 imagery as a geometric skeleton and incorporating DEM constraints, we achieved a meter-level positioning accuracy of 4.68 m (RMSE XY) for mountainous areas and 5.22 m for plain areas.

Validation Conditions and Dependencies: The results demonstrate that the method efficiently mitigates high-order scanning distortions in both mountainous (4.68 m) and plain (5.22 m) environments. However, the final accuracy remains highly dependent on the precision of the reference imagery and the vertical quality of the auxiliary DEM.

Limitations and Future Work: A primary limitation is the sensitivity to significant temporal land-use changes between multi-source sensors, which can introduce matching outliers in rapidly developing urban areas. Future research will focus on integrating multi-temporal robust filtering to further enhance reliability in dynamically changing landscapes.

## Figures and Tables

**Table 1:** Joint Adjustment Positioning Accuracy Statistics (Unit: m).

Image ID	Check Points	RMSE X	RMSE Y	RMSE XY
GF2_3867	845	2.22	2.67	3.48
GF2_3866	1306	3.22	1.88	3.73
GF2_9687	1178	2.37	2.75	3.63
GF2_9689	1045	2.81	2.25	3.6
ZY3_2374	2818	3.26	3.16	4.54
ZY3_7918	2678	4.2	2.49	4.88
Overall		3.08	2.56	4.01

**Table 2:** Edge Matching Accuracy Between Images (Unit: m).

Image Pair		RMSE X	RMSE Y	RMSE XY
GF2_3867	GF2_9687	1.24	1.13	1.68
GF2_3867	GF2_3866	0.50	0.50	0.70
GF2_3867	ZY3_2374	2.67	1.15	2.91
GF2_9687	GF2_9689	0.94	0.52	1.07
GF2_9687	GF2_3866	1.12	0.96	1.48
GF2_9687	ZY3_2374	2.93	1.44	3.27
GF2_9687	ZY3_7918	2.86	1.45	3.20
GF2_9689	GF2_3866	1.36	1.24	1.84
GF2_9689	ZY3_2374	2.77	1.45	3.13
GF2_9689	ZY3_7918	3.03	1.34	3.31
GF2_3866	ZY3_2374	2.52	1.83	3.11
GF2_3866	ZY3_7918	2.73	1.47	3.10
ZY3_2374	ZY3_7918	1.40	1.20	1.85
Overall				2.52

**Table 3:** Original Direct Positioning Accuracy Statistics for Mountainous Areas (Unit: m).

Image ID	Check Points	RMSE X	RMSE Y	RMSE XY
2_1_1	2266	80.38	2.39	80.41
2_1_2	2578	80.3	2	80.33
2_1_3	2687	84.24	3.19	84.3
…	…	…	…	…
2_2_6	2601	82.93	1.82	82.95
2_4_4	2441	83.68	2.3	83.71
2_3_1	2359	83.55	2.44	83.58
Overall		82.13	3.50	82.22

**Table 4:** Original Direct Positioning Accuracy Statistics for Plain Areas (Unit: m).

Image ID	Check Points	RMSE X	RMSE Y	RMSE XY
2_5_8	2321	77.22	3.09	77.28
2_6_8	2524	78.18	3.53	78.26
2_6_7	2259	79.57	4.27	79.69
…	…	…	…	…
2_7_2	2263	78.62	2.82	78.68
2_7_3	2419	79.65	2.97	79.71
2_7_4	2249	80.09	2.34	80.12
Overall		78.78	3.45	78.87

**Table 5:** Collaborative Positioning Accuracy Statistics for Mountainous Areas (Unit: m).

Image ID	Check Points	RMSE X	RMSE Y	RMSE XY
2_1_1	2576	4.45	4.92	6.64
2_1_2	2614	5.51	4	6.81
2_1_3	2652	3.61	2.38	4.33
…	…	…	…	…
2_2_6	2593	1.7	2.65	3.15
2_4_4	2393	2.07	1.66	2.65
2_3_1	2410	1.77	1.72	2.47
Overall		3.39	3.14	4.68

**Table 6:** Collaborative Positioning Accuracy Statistics for Plain Areas (Unit: m).

Image ID	Check Points	RMSE X	RMSE Y	RMSE XY
2_5_8	2597	3.97	2.72	4.81
2_6_8	2181	4.04	2.02	4.51
2_6_7	2499	3.42	2.06	4
…	…	…	…	…
2_7_2	2593	2515	3.68	4.06
2_7_3	2393	2544	3.73	3.39
2_7_4	2410	2337	3.71	2.81
Overall		4.13	3.07	5.22

## Data Availability

The original contributions presented in this study are included in the article. Further inquiries can be directed to the corresponding author.
